# Grape seed-derived procyanidins alleviate gout pain via NLRP3 inflammasome suppression

**DOI:** 10.1186/s12974-017-0849-y

**Published:** 2017-04-04

**Authors:** Hai-Jiao Liu, Xiu-Xiu Pan, Bing-Qian Liu, Xuan Gui, Liang Hu, Chun-Yi Jiang, Yuan Han, Yi-Xin Fan, Yu-Lin Tang, Wen-Tao Liu

**Affiliations:** 1 Jiangsu Key Laboratory of Neurodegeneration, Department of Pharmacology, Nanjing Medical University, Nanjing, Jiangsu 211166 People’s Republic of China; 2 Department of Pharmacology, China Pharmaceutical University, Nanjing, Jiangsu 211198 People’s Republic of China; 3 Department of Ophthalmology, The First Affiliated Hospital of Nanjing Medical University, 300 Guangzhou Road, Nanjing, Jiangsu 210029 People’s Republic of China; 4 Department of Pharmacy, Sir Run Run Shaw Hospital Affiliated to Nanjing Medical University, Jiangsu, 211166 People’s Republic of China; 5 Jiangsu Province Key Laboratory of Anesthesiology, School of Anesthesiology, Xuzhou Medical University, Xuzhou, Jiangsu 221004 People’s Republic of China

**Keywords:** Procyanidins, Gout pain, Macrophages, NLRP3 inflammasome

## Abstract

**Background:**

Gout is one of the common inflammatory arthritis which affects many people for inflicting unbearable pain. Macrophage-mediated inflammation plays an important role in gout. The uptake of monosodium urate (MSU) crystals by macrophages can lead to activation of NOD-like receptors containing a PYD 3 (NLRP3) inflammasome, thus accelerating interleukin (IL)-1β production. Reactive oxygen species (ROS) promoted development of the inflammatory process through NLRP3 inflammasome. Our study aimed to find a food-derived compound to attenuate gout pain via the specific inhibition of the NLRP3 inflammasome in macrophages.

**Methods:**

CD-1 mice were used to evaluate the degree of pain and the swelling dimension of joints after an intra-articular (IA) MSU injection in the ankle. The murine macrophage cell line Raw 264.7 was used to investigate the effects of procyanidins and the mechanism underlying such effects. Histological analysis was used to measure the infiltration of inflammatory cells. ROS produced from Raw 264.7 cells were evaluated by flow cytometry. Cell signaling was measured by Western blot assay and immunofluorescence.

**Results:**

Procyanidins significantly attenuated gout pain and suppressed ankle swelling. Procyanidins also inhibited MSU-induced activation of the NLRP3 inflammasome and increase of IL-1β. Furthermore, procyanidins decreased ROS levels in Raw 264.7 cells.

**Conclusions:**

Suppression of the NLRP3 inflammasome in macrophages contributes to the amelioration of gout pain by procyanidins.

**Electronic supplementary material:**

The online version of this article (doi:10.1186/s12974-017-0849-y) contains supplementary material, which is available to authorized users.

## Background

As one of the most common forms of inflammatory arthritis, gout is caused by monosodium urate (MSU) crystal deposition in and around joints [1]. Acute symptoms of gout patients include redness, swelling, heat, pain, and even joint functional loss [2]. Gout pain is undoubtedly one of the most serious symptoms and can be extreme, even disabling [3]. Therefore, the development of efficient analgesia for gout pain is of very important clinic significance. Regretfully, current gout pain management is far from satisfactory [4]. Hence, a safer and more potent drug is urgently needed for the treatment of gout pain.

Multiple lines of evidence show that macrophages play important roles in the pathogenesis of gout pain [5]. Once activated, macrophages release numerous inflammatory cytokines, including tumor necrosis factor (TNF)α, interleukin (IL)-1β, and IL-6 [6]. Notably, a persistent overexpression of these proinflammatory cytokines exerts algesic effects by acting on nociceptors, exacerbating pain [7]. Furthermore, inflammation in and around the joint can recruit more inflammatory cells, causing edema and joint injury.

Among the inflammatory factors mentioned above, particular attention has been paid to IL-1β in gout pain and inflammation [8, 9]. Previous studies have demonstrated that MSU-induced inflammatory and hypernociceptive responses are greatly decreased in mice deficient in IL-1β or the IL-1 receptor (IL-1R). In addition, the blockade of IL-1R-mediated signaling also weakens the inflammatory and hypernociceptive responses resulting from MSU crystals [10–12].

Studies have shown that NOD-like receptors containing a PYD 3 (NLRP3) inflammasomes play a critical role in MSU-induced IL-1β secretion in macrophages [5]. There are four kinds of inflammasomes, namely NLRP1, NLRP3, NLRC4, and AIM2. The NLRP3 inflammasomes play an important role in macrophages by cleaving pro-IL-1β into mature IL-1β [13, 14]. It has been found that MSU-induced inflammation and pain responses are significantly reduced in NLRP3-deficient mice [15].

Recently, it has been reported that cherry intake is associated with significant decreases of gout attacks [16]. Cherry contains high levels of procyanidins, which have anti-inflammatory and anti-oxidant properties [17, 18]. Studies also showed that the consumption of procyanidin-rich foods can lower the incidence of inflammatory diseases, including metabolic syndrome and atherosclerosis [19]. Moreover, we have previously reported that procyanidins can strongly inhibit the morphine-induced activation of NLRP3 inflammasomes in microglia [20]. On that basis, we hypothesized that procyanidins, a safe and effective natural product, might attenuate gout pain by inhibiting NLRP3 inflammasome activation and IL-1β maturation in macrophages.

## Methods

### Animals and model

Adult CD-1 mice (18–22 g) were provided by the Experimental Animal Center at Nanjing Medical University, Nanjing, China. Animals were housed five to six per cage under pathogen-free conditions with soft bedding under controlled temperature (22 ± 2 °C) and a 12-h light/dark cycle (lights on at 8:00 a.m.). The animals were allowed to acclimate to these conditions for at least 2 days before starting experiments. For each group of experiments, the animals were matched by age and body weight. All surgeries were done under anesthesia induced by chloral hydrate. 0.5 mg of MSU crystals in 10 μL of PBS was injected intra-articularly in one ankle joint. Mechanical hyperalgesia, observed as an increase in nociceptive response, was assessed by Von Fray assay [21, 22] and expressed as mechanical paw withdrawal threshold (*g*). Edema formation was described as the circumstance difference (Δmm) between the basal value and the test value.

### Reagents

Procyanidins were purchased from Zelang Pharmaceutical Co. Ltd. (Nanjing, China). The purity of procyanidins was more than 95%. Procyanidins contained 1.1% monomeric, 34.2% dimeric, 24.9% trimeric, 6.7% tetrameric (totally 66.9% oligomeric procyanidins), and 33.1% polymeric procyanidins. IL-1β was from R&D Systems (Minneapolis, MN, USA). Antibodies for caspase-1 and NLRP3 were acquired from Adipogen International (San Diego, CA, USA). Antibody for glyceraldehyde-3-phosphate dehydrogenase (GAPDH) was from Sigma-Aldrich (St. Louis, MO, USA). Antibodies for phosphorylated N-methyl-D-aspartic acid receptor (NR)1 subunit (Ser896), phosphorylated extracellular regulated protein kinase (ERK; Thr202/Tyr204), phosphorylated c-Jun N-terminal kinase (JNK; Thr183/Tyr185), phosphorylated p38 mitogen-activated protein kinase (p38; Tyr182), and c-fos were from Cell Signaling Technology (Beverly, MA, USA). ROS Assay Kit was from KeyGEN (Nanjing, China). Lipopolysaccharide (LPS) and dimethyl sulfoxide (DMSO) were purchased from Sigma-Aldrich (St. Louis, MO, USA). Fetal bovine serum (FBS) was purchased from Gibco, and other cell culture media and supplements were purchased from HyClone (Logan, UT, USA). 3-(4, 5-Dimethyl-2-thiazolyl)-2,5-diphenyl-2H-tetrazolium bromide (MTT) was purchased from Sunshine Biotechnology (Nanjing, China). MSU crystals were prepared by recrystallisation [5, 11] from uric acid (Sigma-Aldrich, St. Louis, MO, USA). MSU crystals were resuspended in phosphate-buffered saline (PBS) by sonication before used. All other reagents were from Sigma-Aldrich (St. Louis, MO, USA).

### Cell preparation and stimulation

Raw 264.7 was maintained in humidified 5% CO_2_ at 37 °C in Dulbecco’s modified Eagle’s medium supplemented with 10% (*v*/*v*) FBS, penicillin (100 U/ml), and streptomycin ( U/ml). 10^5^ cells were plated in 6-well plate overnight and the medium was changed to serum-free medium in the following morning, and then, the cells were treated with LPS (1 μg/ml) with or without procyanidins (1‰ DMSO) for 6 h and were stimulated with MSU crystals (200 μg/ml) for another 6 h. Cell extracts and precipitated supernatants were analyzed by immunoblotting (Additional file 1).

### Western blot

Periarticular tissue of the ankle and the spinal cord segments at L1-L6 were rapidly removed and homogenized in RIPA Lysis Buffer after the animals’ deep anesthesia with chloral hydrate. The protein concentrations were determined by BCA Protein Assay (Thermo Fisher, Waltham, MA), and 30–60 μg of proteins were loaded and separated by SDS-PAGE and electrophoretically transferred onto polyvinylidene fluoride membranes (Millipore Corp., Bedford, MA). The membranes were blocked with 5% bovine serum albumin for 2 h at room temperature, probed with antibodies overnight at 4 °C with the primary antibodies, and then incubated with HRP-coupled secondary antibodies. The primary antibodies used included IL-1β (1:500), p-NR1 (1:1000), p-p38 (1:1000), p-JNK (1:1000), p-ERK (1:1000), GAPDH (1:8000), NLRP3 (1:1000), and caspase-1 (1:1000). The filters were then developed by enhanced chemiluminescence reagents (PerkinElmer, Waltham, MA) with secondary antibodies (Chemicon, Billerica, MA). Data were acquired with the Molecular Imager (Gel DocTM XR, 170-8170) and analyzed with Quantity One-4.6.5 (Bio-Rad Laboratories, Berkeley, CA, USA).

### Immunofluorescence

After deep anesthesia by intra-peritoneal injection of chloral hydrate, the animal was perfused transcardially with normal saline followed by 4% paraformaldehyde in 0.1 M PB, pH 7.4. Then, L4 and/or L5 lumbar segment was dissected out and post-fixed in 4% paraformaldehyde. The embedded blocks were sectioned as 25 μm thick. Sections from each group (five mice in each group) were incubated with rabbit antibodies for c-fos (1:400). Then, the free-floating sections were washed with PBS and incubated with the secondary antibody for 2 h. After washing out three times with PBS, the samples were studied under an immunofluorescence microscope (Zeiss AX10, Germany) for morphologic details of the immunofluorescence staining. Examination was blindly carried out. Images were randomly coded and the fluorescence intensities were analyzed by Image Pro plus 6.0 software (Media Cybernetics Inc., Rockville, MD). The average green fluorescence intensity of each pixel was normalized to the background intensity in the same image.

### Hematoxylin and eosin staining

Mouse joints were quickly removed from deep anesthetized mice by chloral hydrate, fixed in buffered 10% formalin for 24 h, and decalcified for 12 days in 0.5 M EDTA (pH = 8), finally embedded in paraffin [11, 23]. Then, microtome sections (4 μm) were cut and stained with hematoxylin and eosin (H.E.).

### ROS measurement

Raw 264.7 cells were plated in non-tissue-culture-treated six well dishes and stimulated with MSU (200 μg/ml) for 3 h with or without pre-treatment of procyanidins (10 μM) for 20 min. Positive controls of ROS were incubated for 30 min. After the cultivation, supernatant was removed and cells were washed with PBS. Then, the cells were incubated with 10 μM DCFHDA (to measure mitochondria-associated ROS levels) in serum-free DMEM for 0.5 h at 37 °C. After that, cells were washed with warm PBS, removed from plates with cold PBS, and subjected to fluorescence-activated cell sorting (FACS) analysis (Miltenyi MACSQuant Analyzer 10, Germany). The data were analyzed using FlowJo statistical software (Emerald Biotech Co., Ltd.).

### Statistical analyses

SPSS Rel 15 (SPSS Inc., Chicago, IL) was used to conduct all the statistical analyses. Alteration of expression of the proteins detected and the behavioral responses were tested with one-way ANOVA and the differences in latency over time among groups were tested with two-way ANOVA. Bonferroni post hoc tests were conducted for all ANOVA models. Results are expressed as mean ± SEM of three independent experiments. Results described as significant are based on a criterion of *p* < 0.05.

## Results

### Procyanidins suppressed MSU-induced NLRP3 inflammasome activation in vitro

**Fig. 1:**
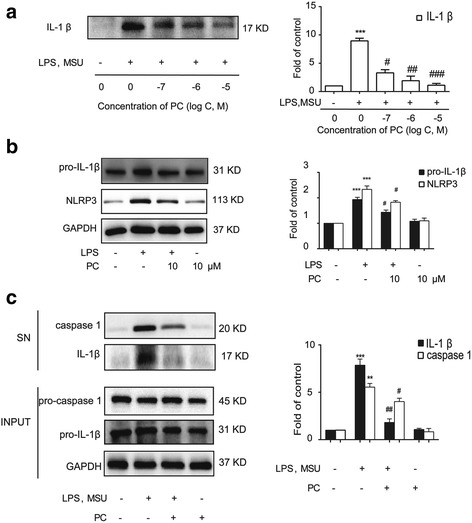
Procyanidins suppressed MSU-induced NLRP3 inflammasome activation in RAW macrophages. **a** The cells were stimulated by LPS (1 μg/ml) for 6 h and then stimulated with MSU crystals for another 6 h. Procyanidins were added 20 min before LPS administration. Western blot samples were prepared from the supernatant (*n* = 4). **b** The cell extracts were collected at 12 h following LPS treatment from Raw 264.7 cells; procyanidins were added 20 min before LPS (*n* = 4). **c** The cells were stimulated by LPS (1 μg/ml) for 6 h and then stimulated with MSU crystals for another 6 h. Procyanidins (10 μM) were added 20 min before LPS. The supernatant (*SN*) and cell lysis fractions (*Input*) of Raw 264.7 cells were collected respectively (*n* = 4). *p < 0.05, **p < 0.01, ***p < 0.001 vs. naive; ^#^ *p* < 0.05, ^##^ *p* < 0.01, ^###^ *p* < 0.001 vs. the MSU-treated group

To study the effects of procyanidins on MSU-induced inflammatory reactions in vitro, MSU crystals were prepared as previously described, and the murine macrophage cell line RAW 264.7 was used [5, 11]. The crystals were found to be stable. Raw 264.7 cells were primed with LPS (1 μg/ml) for 6 h with or without various concentrations of procyanidins 20 min of pre-treatment and then stimulated with MSU crystals (200 μg/ml) for another 6 h. Interestingly, we found that mature IL-1β secretion in the supernatant was inhibited by procyanidins in a dose-dependent manner (Fig. 1a). Since the NLRP3 inflammasome is responsible for IL-1β maturation, we then examined the potential effects of procyanidins on NLRP3 inflammasomes in the cytoplasm. LPS treatment alone for 12 h could trigger the expression of NLRP3 protein and pro-IL-1β, and pre-treatment of procyanidins for 20 min suppressed the increase of NLRP3 and pro-IL-1β (Fig. 1b). Subsets of NLRP3 were able to assemble and oligomerize into a common structure, which collectively activated the caspase-1 cascade, thereby leading to the production of proinflammatory cytokines, especially IL-1β. Procyanidins (10 μM) significantly reduced the release of cleaved caspase-1 and mature IL-1β in Raw 264.7 cells (Fig. 1c).

### Procyanidins suppressed MSU-induced ROS production in vitro

**Fig. 2:**
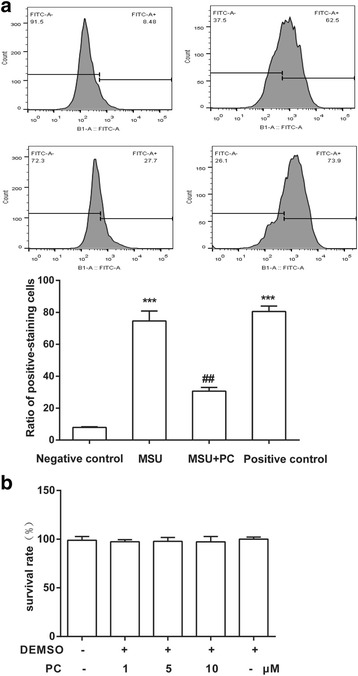
Procyanidins suppressed MSU-induced ROS production in macrophages. **a** The levels of ROS were assessed by calculating the ratio of positive-staining cells among 10,000 cells using flow cytometry. Raw 264.7 cells were primed with procyanidins (10 μM) for 20 min and then stimulated by MSU crystals for 3 h. **b** MTT assay: Raw 264.7 cells were treated with various doses of procyanidins (1, 5, and 10 μM) for 24 h

Studies show that ROS can activate the NLRP3 inflammasome as a second activation signal [24, 25]. Herein, we measured ROS production by flow cytometry in Raw 264.7 cells. The MSU crystals were found to significantly increase the level of ROS compared with the control (negative control). Pre-administration (20 min earlier) with 10 μM procyanidins significantly reduced the MSU-induced production of ROS. The positive control also enhanced ROS production (Fig. 2a). The results of an MTT assay indicated that procyanidins at various doses did not affect cell proliferation (Fig. 2b).

### Procyanidins alleviated gout pain and suppressed MSU-induced ankle swelling in vivo

**Fig. 3:**
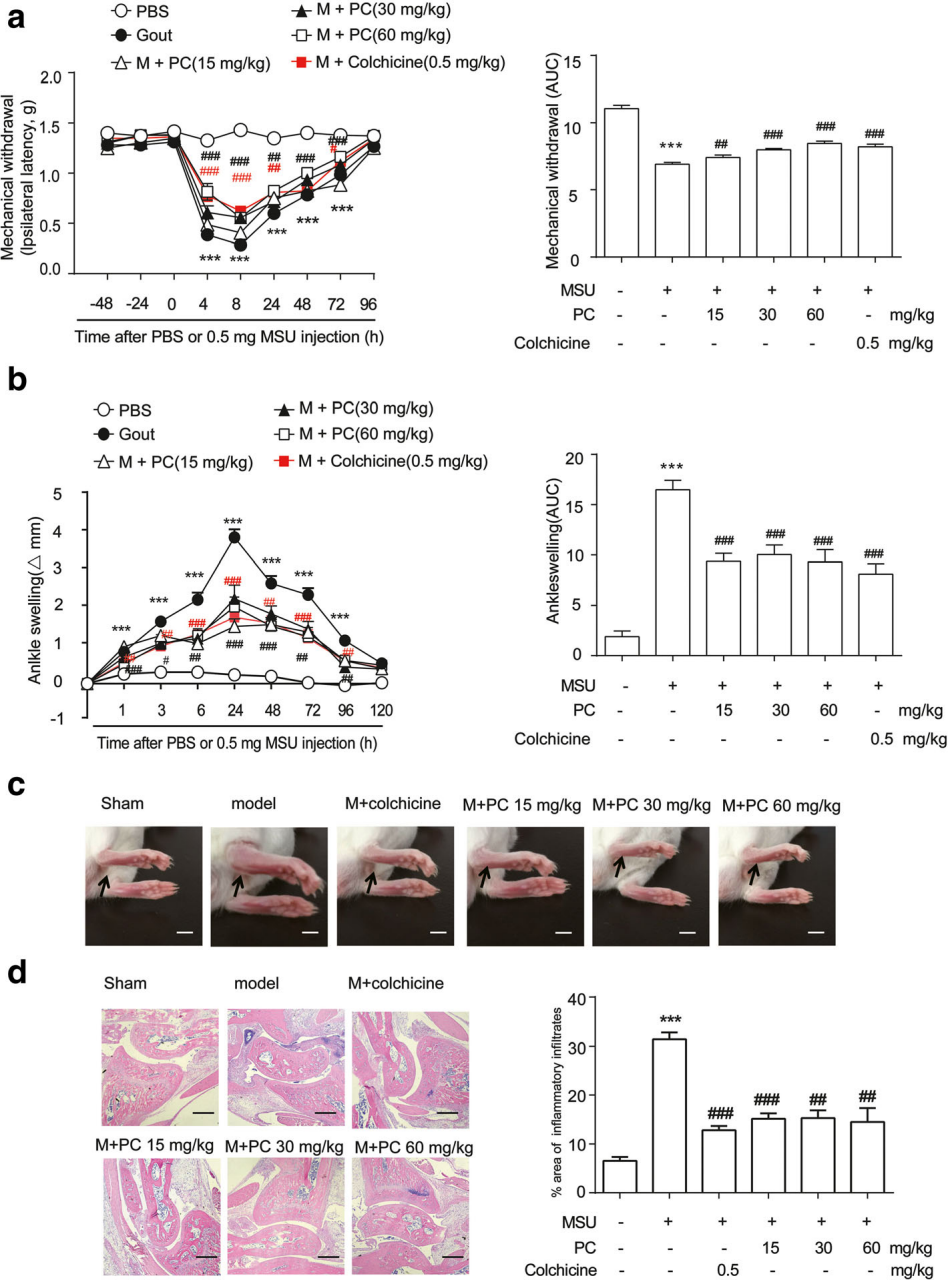
Procyanidins suppressed MSU-induced gout pain in mice. Mice were treated with various doses of procyanidins (PO) 20 min before the injection of MSU crystals (0.5 mg/10 μL). **a** Mechanical allodynia was performed to evaluate the effect of procyanidins (*n* = 8). **b** Time course of changes in MSU-induced ankle swelling (*n* = 8). **c** Representative photographs of mouse ankles at 24 h following MSU injection. *Bar*: 5 mm (**d**) hematoxylin- and eosin-stained sections of the ankle joints obtained 24 h after MSU injection. *Bar*: 100 μm. *p < 0.05, **p < 0.01, ***p < 0.001 vs. normal; ^#^ *p* < 0.05, ^##^ *p* < 0.01, ^###^ *p* < 0.001 vs. the MSU-treated group

We then investigated the role of procyanidins in MSU-induced inflammation in vivo. Mechanical withdrawal decreased to 0.28 g at 8 h after MSU injection. The reduction in MSU-induced pain was significantly reversed to 0.40, 0.55, and 0.56 g by a twice-daily co-administration of procyanidins (15, 30, or 60 mg/kg, PO, respectively) (Fig. 3a). MSU crystals induced ankle swelling, which reached a maximum of 4.3 mm 24 h after the injection. This swelling was significantly attenuated by the listed doses of procyanidins to 1.7, 2.5, and 2.2 mm (Fig. 3b, c, respectively). Histological analysis showed that the MSU crystals significantly increased leukocyte infiltration into the superficial synovium. Co-treatment with procyanidins (15, 30, or 60 mg/kg, PO) inhibited leukocyte infiltration (Fig. 3d).

### Procyanidins inhibited NLRP3 inflammasome activation in vivo

**Fig. 4:**
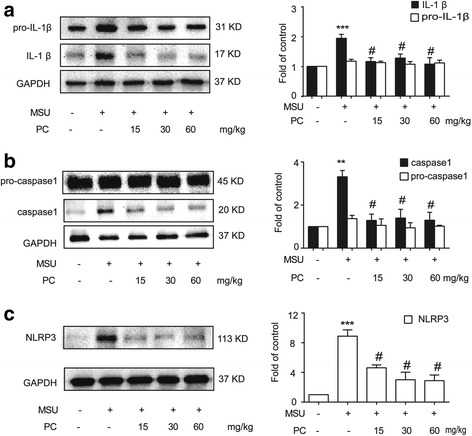
Procyanidins suppressed NLRP3 inflammasome activation in ankle periarticular tissue. Mice were treated with various doses of procyanidins (15, 30, and 60 mg/kg, PO) 20 min before the injection of MSU crystals (0.5 mg/10 μL). Western blot samples were collected 24 h after MSU injection (*n* = 4). Expression of pro- and cleaved IL-1β (**a**), pro- and cleaved caspase-1 (**b**), and NLRP3 (**c**) in ankle periarticular tissue are shown. *p < 0.05, **p < 0.01, ***p < 0.001 vs. normal; ^#^ *p* < 0.05, ^##^ *p* < 0.01, ^###^ *p* < 0.001 vs. the MSU-treated group

Gout results in an increased inflammatory response; in particular, IL-1β is upregulated [26]. Our data showed that an IA injection of MSU crystals (0.5 mg/10 μl) in the ankles of CD-1 mice increased the protein levels of the proinflammatory cytokine IL-1β in the periarticular tissue of the ankle. Procyanidins (20 min before IA injection of MSU crystals, PO) efficiently suppressed the upregulation of proinflammatory cytokines (Fig. 4a). Western blot analysis revealed that procyanidins significantly reduced the increased protein levels of caspase-1 and NLRP3 (Fig. 4b, c).

### Procyanidins inhibited the phosphorylation of NR1, p38, and ERK and the activation of c-fos in the spinal cord

**Fig. 5:**
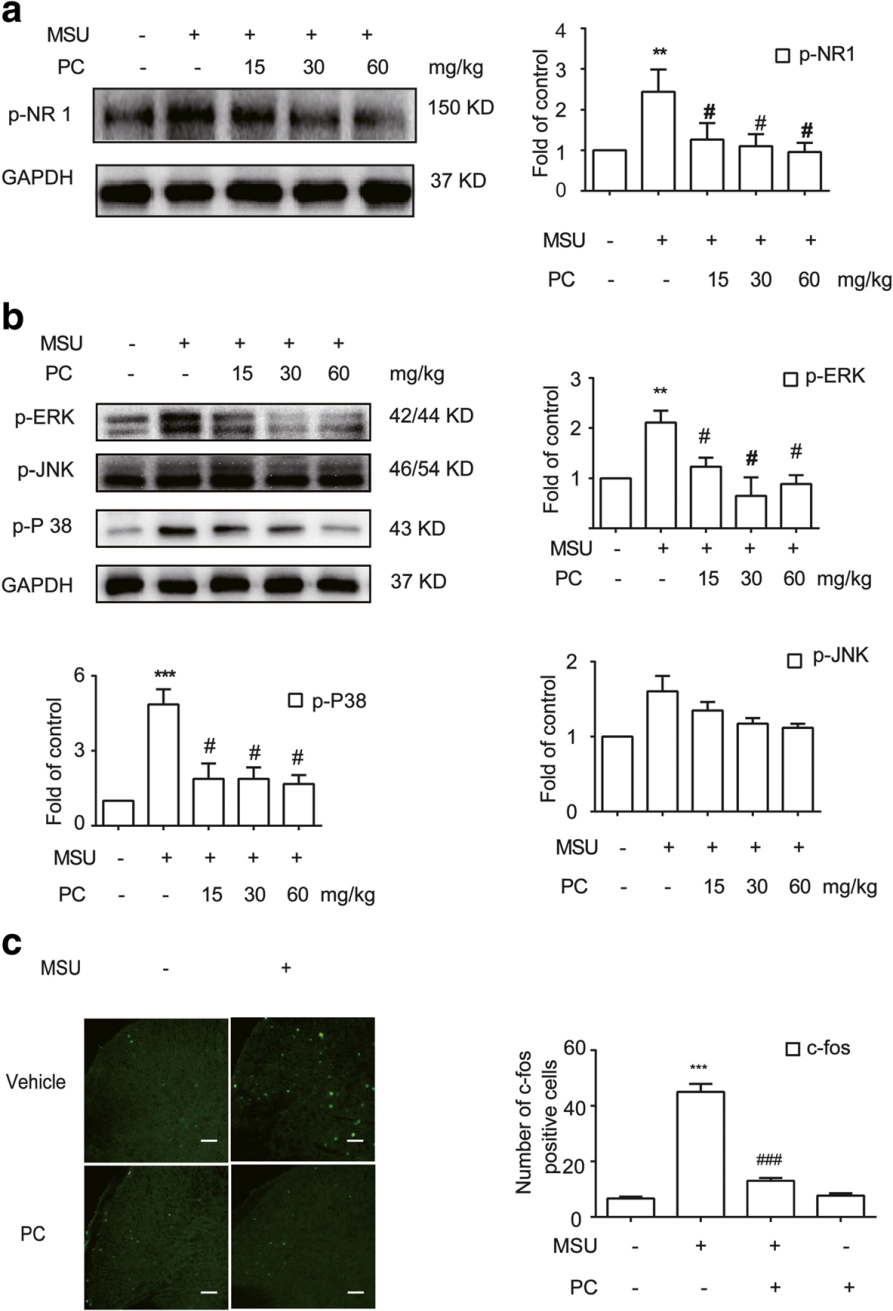
Procyanidins inhibited central sensitization. **a** Phosphorylation of NMDA receptors (*n* = 4). **b** Phosphorylation of p38, ERK, and JNK (*n* = 4). **c** Immunofluorescence analysis of c-fos in the dorsal horn of the spinal cord (*n* = 5). The quantification of c-fos immunofluorescence is represented as the number of c-fos-positive cells in the superficial dorsal horns. *Bar*: 80 μm *p < 0.05, **p < 0.01, ***p < 0.001 vs. normal; ^#^ *p* < 0.05, ^##^ *p* < 0.01, ^###^ *p* < 0.001 vs. the MSU-treated group

Studies have shown that activated macrophages can release proinflammatory cytokines, such as IL-1β and TNF-α, which lead to the phosphorylation of MAPKs and NMDA, in turn resulting in central sensitization and hyperalgesia [27]. We investigated the effects of procyanidins on MSU-induced central sensitization in vivo. Western blot analysis revealed that phosphorylated NR1 was upregulated in the spinal cords of mice treated with MSU crystals in the ankles (Fig. 5a). However, procyanidins suppressed this increase in NR1 phosphorylation. The compounds also inhibited the phosphorylation of p38 and ERK (Fig. 5b). Furthermore, immunofluorescence analysis showed that the expression of c-fos protein, which is encoded by an immediate-early gene rapidly expressed in neurons after a noxious stimulus, was increased in the dorsal horn of the spinal cord after MSU injection; this increase was then suppressed by procyanidins (Fig. 5c).

## Discussion

In this study, we found that the clinically used health products, procyanidins, had a significant inhibitory effect on MSU-induced NLRP3 activation. Moreover, the development of MSU-induced pain and ankle swelling was markedly attenuated by procyanidin administration.

Objectives for gout treatment include managing the symptoms of acute attacks and preventing further attacks by reducing uric acid levels in the blood. The most commonly used therapies for acute gout in general practice include the use of non-steroidal anti-inflammatory drugs (NSAIDs), colchicine, and corticosteroids. Although these drugs have certain therapeutic effects, they present serious side effects, such as liver and kidney damage and severe gastrointestinal reactions [3, 4, 28]. Moreover, the treatment of gout is often a long-term process. Therefore, searching for safer compounds is a very attractive strategy. Recently, cherries have attracted considerable attention and interest as a promising candidate for the prevention and management of gout [29]. Cherries are rich in procyanidins, which are natural anti-oxidants and are currently recognized as the most effective free radical scavengers [17, 30, 31]. Procyanidins are found in grape seeds, cranberries, black wolfberry, and other sources and are therefore widely available. The oral LD50 values of procyanidins are over 4000 mg/kg in mice, indicating a high level of safety. In our study, the high dose of 60 mg/kg was used in mice, which is equivalent to 300 mg per day in humans. Thus, the dose applied in our experiment is reasonably believed to be safe.

The intra-articular injection of MSU crystals induces the onset of pain-like behavior in mice [10, 32]. In accordance with the findings of a previous study, the mechanical pain threshold of mice decreased obviously and allodynia was induced after an intra-articular ankle injection of MSU crystals; this effect reached a maximum 8 h after injection and lasted 72 h in our study. Moreover, procyanidin (15, 30, 60 mg/kg, PO) administration strongly inhibited MSU-induced gout pain in a dose-dependent manner, and the effect of procyanidin (30 mg/kg, PO) administration worked as well as 0.5 mg/kg of colchicine.

Evidence presented by Laurent L. Reber et al. showed that maximal ankle swelling is reached within 24 h of an intra-articular injection of MSU crystals in one mouse ankle [11]. These results are consistent with our findings that ankle swelling reached a maximum at 24 h and the ankle circumference increased to 4.3 mm. Procyanidin (15, 30, 60 mg/kg, PO) administration decreased the circumference of the ankle to 1.7, 2.5, and 2.2 mm, respectively, after 24 h. The results of a histological assay confirmed that joint inflammation occurred in the joint space after injecting MSU crystals [11, 33]. A model group showed a local infiltration of inflammatory cells as well as acute inflammation and tissue proliferation in the ankle joint. However, a significant reduction in these pathological changes was found in histological sections prepared from procyanidin-pretreated acute gout mice.

The MSU-induced production of IL-1β, which is mediated by the NLRP3 inflammasome activation in macrophages, is a key pathological mechanism underlying gout [5, 34, 35]. NLRP3 inflammasome activation involves a two-step process. First, signal 1, also known as the priming signal, activates the NF-κB pathway, leading to the upregulation of pro-IL-1β and NLRP3 protein levels. Second, signal 2 is transduced by various pathogen-associated molecular patterns (PAMPs) and damage-associated molecular patterns (DAMPs). Currently, several molecular mechanisms have been suggested for NLRP3 activation, including potassium efflux, lysosomal destabilization, and ROS generation [36, 37]. Our study revealed that procyanidins significantly decreased NLRP3 expression. In addition, it has been proved that mIL1 Trap prevents and suppresses MSU-induced hyperalgesia and inflammation in a mouse model of acute gouty ankle arthritis [10]. Consistent with these results, we found that procyanidins can markedly decrease caspase-1 and IL-1β levels, which greatly aids the alleviation of pain and ankle swelling.

We then investigated the possible mechanisms underlying NLRP3 inflammasome inhibition by procyanidins. As mentioned previously, MSU crystals induce the dissociation of TXNIP from thioredoxin in a ROS-sensitive manner, enabling it to bind to NLRP3 [38, 39]. Evidence exists that ROS scavengers can block inflammasome activation [40]. In accordance with this notion, we found that MSU crystals induced a very significant increase of ROS in macrophages and that procyanidins markedly inhibit MSU-induced ROS production. Our results suggest that procyanidins may inhibit NLRP3 inflammasome activation by scavenging ROS.

In addition to studying peripheral joints, we also assessed indicators of central sensitization. The *N*-methyl-*D*-aspartate receptor (NMDAR) activation is an essential step in both starting and maintaining activity-dependent central sensitization. NMDAR antagonists prevent nociceptive neuron hyperexcitability, which is induced by nociceptor conditioning inputs. NR1 conditional deletion even abolishes NMDA synaptic inputs and acute activity-dependent central sensitization. Moreover, NMDAR activation leads to a rapid increase of [Ca^2+^], which activates protein kinase C(PKC) and calmodulin-dependent protein kinase II (CaMKII), subsequently leading to the activation of c-fos [41, 42]. Intra-cellular pathways including PLC/PKC, phosphatidylinositol-3-kinase (PI3K), and the mitogen-activated protein kinase (MAPK) can sustain central sensitization. Among these pathways, the phosphorylation of MAPK family proteins, especially p38, has the greatest influence on pain progression. p38 phosphorylation results in the synthesis and release of numerous inflammatory mediators, including IL-1β [43, 44]. Our study showed that MSU crystals significantly increased the levels of p-NR1, c-fos, and p-MAPK in the spinal cord, suggesting that NR1, c-fos, and MAPK may also be involved in gout pain. Procyanidins significantly inhibited the expression of p-NR1, c-fos, and p-MAPK, indicating their potential effect on the chronicity of gout pain. Since our study focused on acute pain, we will conduct further research on the mechanisms underlying chronic pain in the future.

## Conclusions

In this study, we report a biological mechanism that can suppress inflammation and ameliorate gout pain via NLRP3 inflammasome suppression in macrophages. Our results demonstrated that procyanidins scavenged oxygen-free radicals, suppressed NLRP3 inflammasome activation, and inhibited the production of inflammatory cytokines and inflammatory infiltration. Procyanidins represent potential candidate drugs for the management of gout in the clinic.

## Additional file

Additional file 1: Figure S1. The Raw 264.7 cells were treated with or without LPS (1 μg/ml) for 6 h and then stimulated with MSU crystals for another 6 h. **Figure S2.** The levels of ROS were assessed by calculating the ratio of positive-staining cells among 10,000 cells using flow cytometry. Raw 264.7 cells were treated with procyanidins (10 μM) for 20 min and then stimulated by MSU crystals for 3 h. (DOCX 698 kb)

## Abbreviations

BSA: Bovine serum albumin
CaMKII: Calmodulin-dependent protein kinase II
DCFHDA: 2′,7′-Dichlorofluorescin diacetate
DMEM: Dulbecco’s modified Eagle’s medium
DMSO: Dimethyl sulfoxide
ERK: Extracellular regulated protein kinases
FACS: Fluorescence-activated cell sorting
FBS: Fetal bovine serum
FITC: Fluorescein isothiocyanate
GAPDH: Glyceraldehyde-3-phosphate dehydrogenase
HE: Hematoxylin and eosin
IA: Intra-articular
IL-1β: Interleukin-1β
IL-6: Interleukin-6
JNK: c-Jun N-terminal kinase
LPS: Lipopolysaccharide
MAPK: Mitogen-activated protein kinase
MSU: Monosodium urate
MTT: 3-(4,5-Dimethyl-2-thiazolyl)-2,5-diphenyl-2-*H*-tetrazolium bromide
NF- κB: Nuclear factor- κB
NLRP3: NOD-like receptors containing a PYD 3
NMDAR-NR1: *N*-Methyl-*D*-aspartic acid receptor NR1
NSAIDs: Non-steroidal anti-inflammatory drugs
PB: Phosphate buffer
PBS: Phosphate-buffered saline
PC: Procyanidins
PI3K: Phosphatidylinositol-3-kinase
PKC: Protein kinase C
PLC: Phospholipase C
RIPA: Radio-immunoprecipitation assay
ROS: Reactive oxygen species
SDS-PAGE: Sodium dodecyl sulfate-polyacrylamide gel electrophoresis
TNF-α: Tumor necrosis factor-α
TXNIP: Thioredoxin-interacting protein
