# Supplementary material for: Grape seed-derived procyanidins alleviate gout pain via NLRP3 inflammasome suppression
Source: J Neuroinflammation. 2017 Apr 4;14:74. doi: 10.1186/s12974-017-0849-y (PMC5381065; doi:10.1186/s12974-017-0849-y)
Supplement: Additional file 1: Figure S1. — The Raw 264.7 cells were treated with or without LPS (1 μg/ml) for 6 h and then stimulated with MSU crystals for another 6 h. Figure S2. The levels of ROS were assessed by calculating the ratio of positive-staining cells among 10,000 cells using flow cytometry. Raw 264.7 cells were treated with procyanidins (10 μM) for 20 min and then stimulated by MSU crystals for 3 h. (DOCX 698 kb) [file 12974_2017_849_MOESM1_ESM.docx]

**Grape seed derived procyanidins alleviate gout pain via NLRP3 inflammasome suppression**

Hai-Jiao Liu^1,2*^, Xiu-Xiu Pan^1*^, Bing-Qian Liu^3^, Xuan Gui^4^, Liang Hu^1^, Chun-Yi Jiang^1^, Yuan Han^5^, Yi-Xin Fan^2^, Yu-Lin Tang^4^, Wen-Tao Liu^1#^

^1^Jiangsu Key Laboratory of Neurodegeneration, Department of Pharmacology, Nanjing Medical University, Nanjing, Jiangsu 211166, People’s Republic of China.

^2^Department of Pharmacology, China Pharmaceutical University, Nanjing, Jiangsu, 211198, People’s Republic of China.

^3^Department of Ophthalmology, The First Affiliated Hospital of Nanjing Medical University, 300 Guangzhou Road, Nanjing, Jiangsu 210029, People’s Republic of China.

^4^Department of Pharmacy, Sir Run Run Shaw Hospital Affiliated to Nanjing Medical University, Jiangsu 211166, People’s Republic of China.

^5^Jiangsu Province Key Laboratory of Anesthesiology, School of Anesthesiology, Xuzhou Medical College, Xuzhou, Jiangsu 221004, People’s Republic of China.

^*^Equal contributors

^#^Corresponding author: painresearch@njmu.edu.cn

Co-authors' email addresses

Hai-Jiao Liu: 442763510@qq.com; Xiuxiu Pan: 1160028206@qq.com; Bing-Qian Liu: 13151370243@qq.com; Xuan Gui: 115650770@qq.com; Liang Hu: lianghu@njmu.edu.cn; Chun-Yi Jiang: jcy@njmu.edu.cn; Yuan Han: 374198873@qq.com; Yi-Xin Fan: 1026912119@qq.com; Yu-Lin Tang: 3202245011@qq.com; Wen-Tao Liu: [painresearch@njmu.edu.cn](mailto:painresearch@njmu.edu.cn)

**Supplementary Data**

**
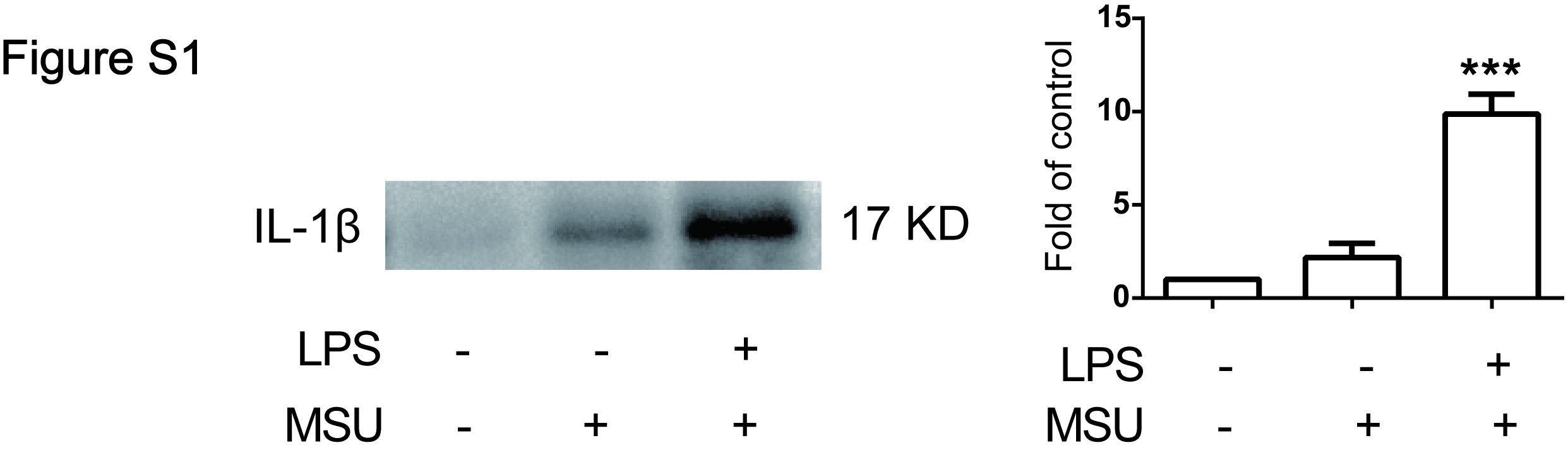
**

Figure S1. The RAW 264.7 cells were treated with or without LPS (1 μg/ml) for 6 hours and then stimulated with MSU crystals for another 6 hours.

**Figure S2**

**
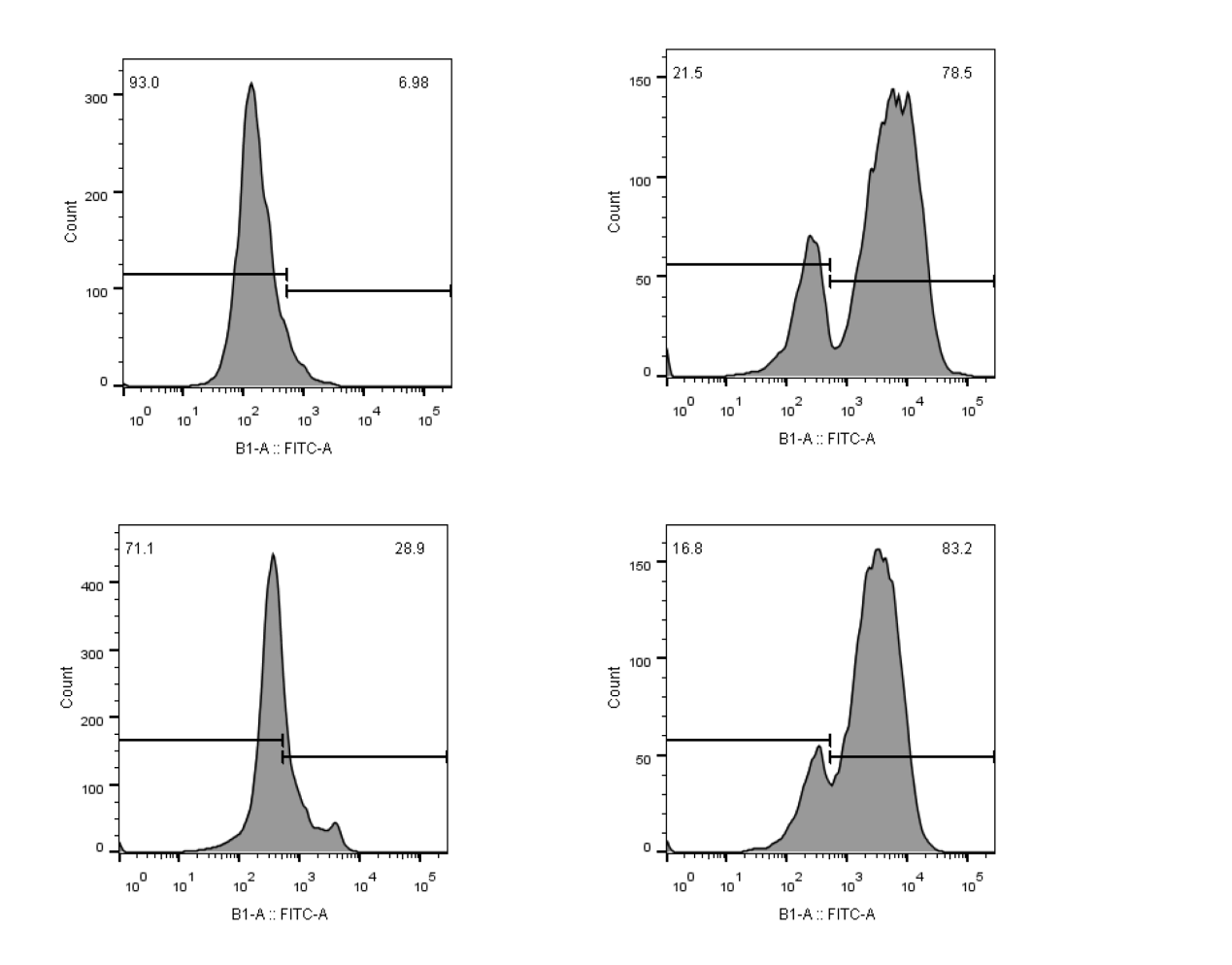
**

**
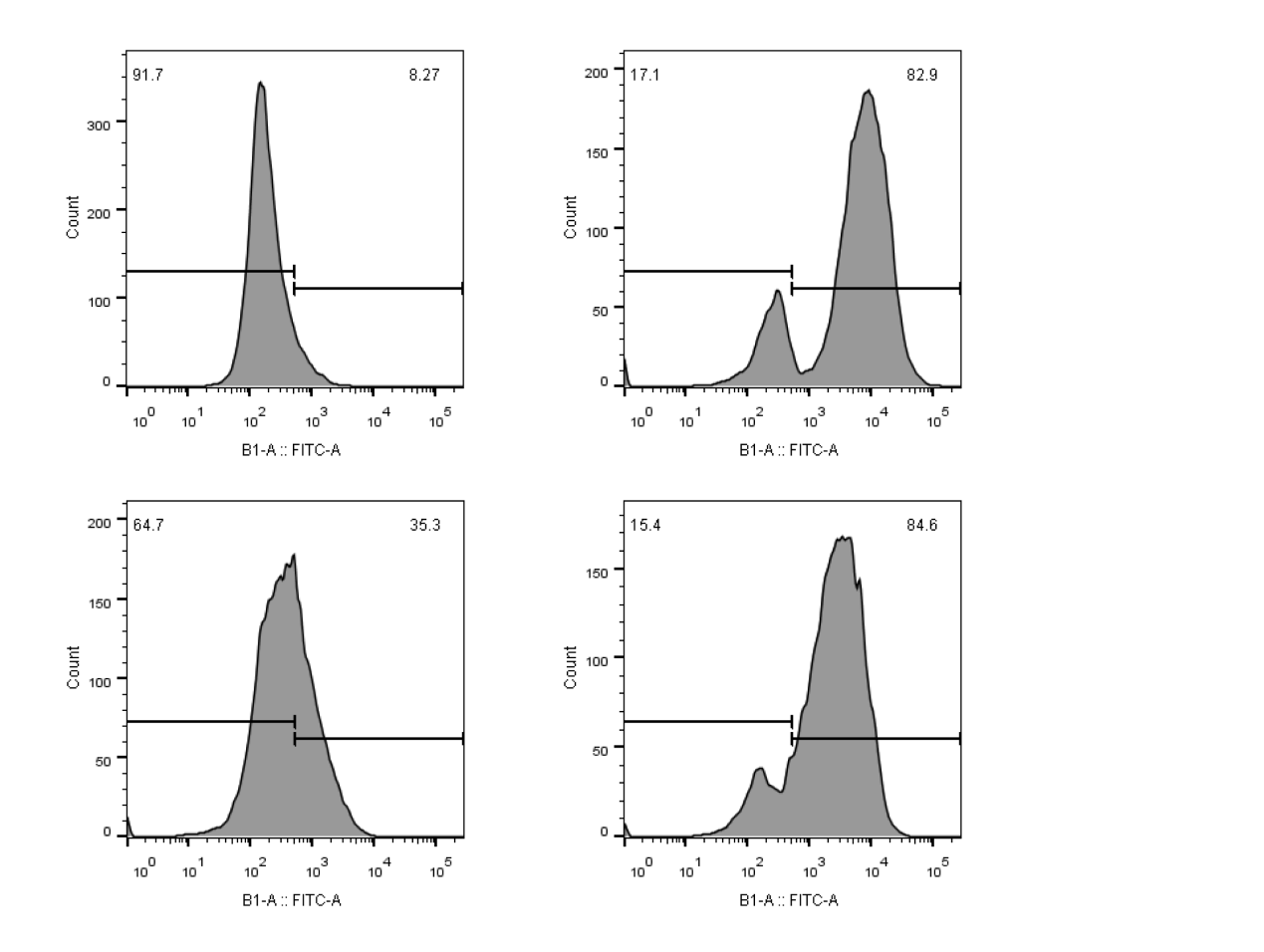
**

Figure S2. The levels of ROS were assessed by calculating the ratio of positive-staining cells among 10,000 cells using flow cytometry. RAW 264.7 cells were treated with procyanidins (10 μM) for 20min then stimulated by MSU crystals for 3 hours.
